# Structural heterogeneity of the mammalian polycomb repressor complex in immune regulation

**DOI:** 10.1038/s12276-020-0462-5

**Published:** 2020-07-07

**Authors:** Seok-Jin Kang, Taehoon Chun

**Affiliations:** Department of Biotechnology, College of Life Sciences and Biotechnology, Korea University, Seoul, 02841 Republic of Korea

**Keywords:** Immunogenetics, Haematopoiesis

## Abstract

Epigenetic regulation is mainly mediated by enzymes that can modify the structure of chromatin by altering the structure of DNA or histones. Proteins involved in epigenetic processes have been identified to study the detailed molecular mechanisms involved in the regulation of specific mRNA expression. Evolutionarily well-conserved polycomb group (PcG) proteins can function as transcriptional repressors by the trimethylation of histone H3 at the lysine 27 residue (H3K27me3) and the monoubiquitination of histone H2A at the lysine 119 residue (H2AK119ub). PcG proteins form two functionally distinct protein complexes: polycomb repressor complex 1 (PRC1) and PRC2. In mammals, the structural heterogeneity of each PRC complex is dramatically increased by several paralogs of its subunit proteins. Genetic studies with transgenic mice along with RNA-seq and chromatin immunoprecipitation (ChIP)-seq analyses might be helpful for defining the cell-specific functions of paralogs of PcG proteins. Here, we summarize current knowledge about the immune regulatory role of PcG proteins related to the compositional diversity of each PRC complex and introduce therapeutic drugs that target PcG proteins in hematopoietic malignancy.

## Immunology: Gene repressors regulating immunity offer cancer drug targets

Protein complexes that suppress gene activity by remodeling chromatin, the substance that contains most of a cell’s DNA, play a critical role in regulating the immune system and provide a therapeutic target for treating blood cancers. Seok-Jin Kang and Taehoon Chun from Korea University in Seoul, South Korea, review how polycomb group proteins, best known for their function in embryonic development, also contribute to the formation of immune cells from blood stem cell precursors. Studies with stem cells and cancer cells have begun to reveal many targets of these proteins, and drug companies are evaluating candidate agents directed against some polycomb group proteins in patients with lymphoma and other cancers. More comprehensive profiling of protein function across a broad range of immune cell types could reveal new targets for additional diseases associated with immune dysfunction.

## Introduction

In eukaryotes, the alteration of chromatin structure is one of the main methods for modifying cell phenotypes by regulating specific DNA replication and mRNA transcription^1^. In addition to DNA methylation, changing the properties of certain amino acid residues at histones is a major method for modifying the structure of chromatin. The enzymes involved in the acetylation, methylation, ubiquitination, and phosphorylation of histones have been identified and extensively studied to define the biological function of each enzyme^2^. Many studies have provided evidence that histone modification plays a decisive role in cell fates such as carcinogenesis, differentiation, proliferation, and senescence^3^.

Polycomb group (PcG) proteins were originally identified from fruit flies. They are well conserved from invertebrates to mammals during evolution. PcG proteins can act as transcriptional repressors by inhibiting the mRNA transcription of specific gene loci through the trimethylation or monoubiquitination of histones H3 and H2A^4^. To initiate and maintain such chromatin modification, two distinct protein complexes, polycomb repressor complex 1 (PRC1) and PRC2, work in coordination with each other. PRC2 exhibits methyltransferase activity to add methyl functional groups to specific amino acid residues of histone H3, while PRC1 exhibits E3 ubiquitin-ligase activity to modify the structure of histone H2A^4,5^. Mammalian PRC complexes display structural plasticity because the existence of several paralogs of PcG subunit proteins^6^. In particular, more than 100 different types of mammalian PRC1 complexes may exist based on a simple combinatorial algorithm^7^.

Although recent progress in biochemical and molecular analyses involving transgenic animal techniques has revealed the functional importance of the core subunit of the PcG proteins that regulate mRNA expression through histone modifications, how each of the paralogs of PcG subunit protein interact with each other to orchestrate the fine tuning of chromatin structure remains elusive. In this review, we summarize current knowledge about the immune regulatory role of PcG proteins related to the compositional diversity of each PRC complex. We also introduce therapeutic drugs that target PcG proteins.

## Structural heterogeneity related to the function of PcG proteins

PcG genes were initially identified as genes involved in the regulation of homeotic gene expression, critical for the body axis plan and segment development in fruit flies^8^. PcG proteins are present in plants, nematodes, and metazoan species from flies to mammals, indicating that these proteins are well-conserved transcriptional repressors via the modification of chromatin structure during evolution^9^. Each PcG protein is a subunit of multiprotein complexes categorized by two different functional groups: PRC1 and PRC2^10^.

**Fig. 1:**
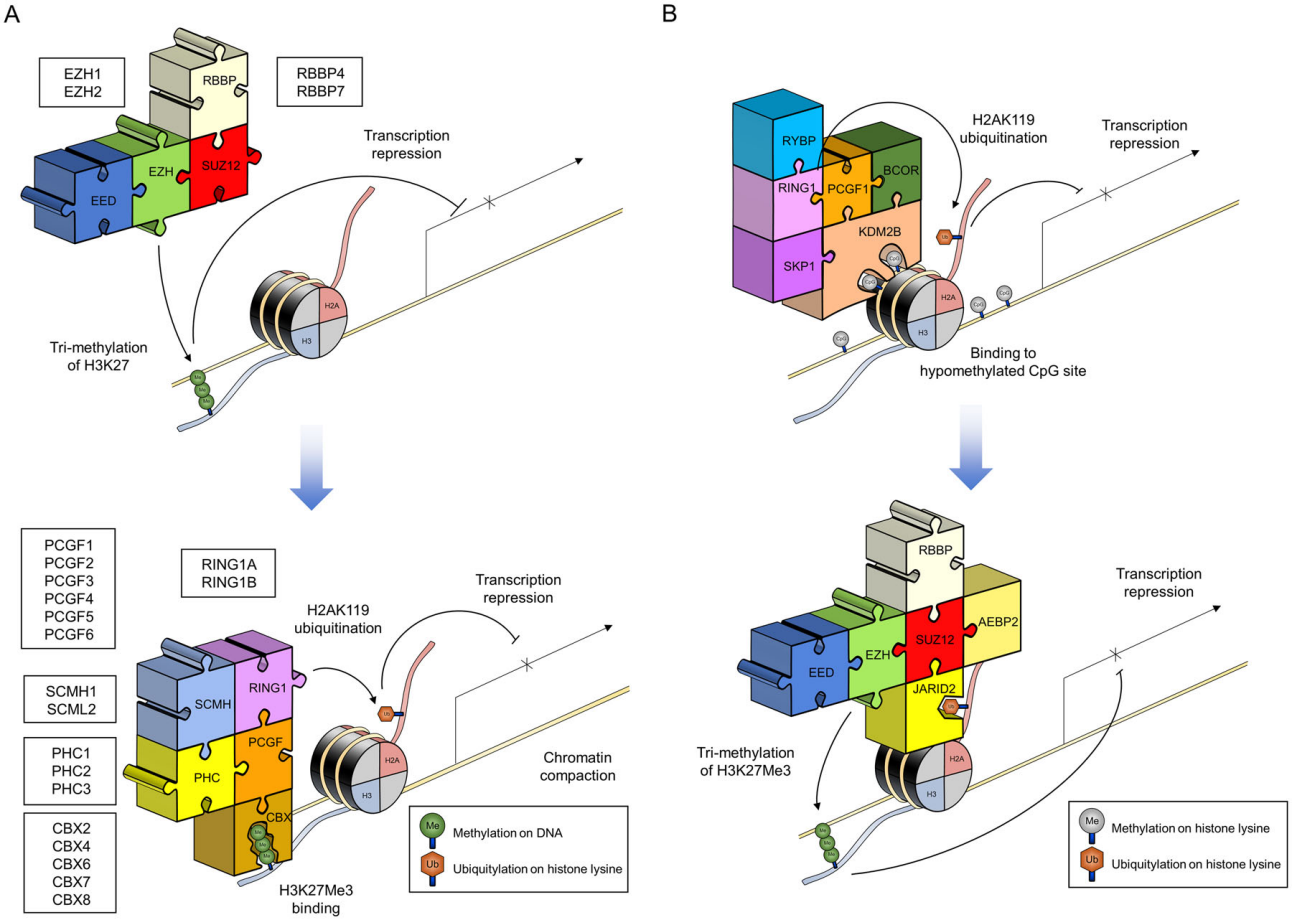
The repressive mechanism of specific mRNA transcription by PcG proteins through the modification of chromatin structure. Schematic representation of transcriptional repression by PcG proteins according to the ‘hierarchical repressive model’ (a) and the ‘reverse-hierarchical repressive model’ (**b**). **a** Core subunits of PRC2 (EED, EZH, SUZ12, RBBP) recognize and repress a target locus by introducing H3K27me3. The CBX subunit of canonical PRC1 (PRC1.2 and PRC1.4) then recognizes the H3K27me3 tag, and canonical PRC1 further represses the target locus by introducing H2AK119. **b** The KDM2B subunit of noncanonical PRC1 (PRC1.1) recognizes CpG, and PRC1.1 represses the target locus by introducing H2AK119. The JARID2 subunit of PRC2.2 then recognizes the H2AK119 tag, and PRC2.2 further represses the target locus by introducing H3K27me3.

Embryonic ectoderm development (EED), suppressor of zeste (SUZ)12, and enhancer of zeste homolog (EZH) are the catalytic core subunits of PRC2. Since EZH has two paralogs (EZH1 and EZH2), two structural variants are found in the catalytic core of PRC2 (Fig. 1a)^11^. EZH2 is the enzymatic subunit of the PRC2 complex, which acts as an S-adenosyl-l-methionine (SAM)-dependent histone methyltransferase via the mono-, di-, or trimethylation of lysine 27 residue at histone H3 (H3K27me1, H3K27me2, or H3K27me3) (Fig. 1 and Table 1)^11–16^. EZH1 also acts as a methyltransferase with reduced enzyme activity compared to EZH2^10^. The SET domain of EZH1 or EZH2, which contains the catalytic core and SAM-binding site, is indispensable for their methyltransferase activity. However, purified EZH1 or EZH2 monomers alone are unable to efficiently exert enzyme activity in vitro because they must bind with two other noncatalytic subunit proteins, SUZ12 and EED (Fig. 1, Tables 1 and 2)^9,11,12,17–21^. SUZ12 contains a zinc-finger domain that can bind to DNA or RNA and facilitate protein–protein interactions^22^. EED contains WD40 repeats that can putatively bind to H3K27me3 (Table 2)^23^. The fourth member of the PRC2 core subunit is retinoblastoma-binding protein 4 (RBBP4) (NURF55) or RBBP7 (Fig. 1, Tables 1 and 2)^9,12,18,19,24,25^. Whether RBBP 4/7 is included in the catalytic core of PRC2 is still controversial because RBBP 4/7 activity is not required for the catalytic activity of PRC2 in vitro^26^. However, RBBP 4/7 also contains WD40 domains that can bind to histones and facilitate the catalytic activity of PRC2 in vivo^26^.

**Table 1:** Each subunit of canonical and noncanonical polycomb complexes in mammals.

PRC	Core subunits	Classification	Other subunits	Reference
PRC2.1	EZH, EED, SUZ12, RBBP	N.D.	PCL, EPOP (C17orf96), LCOR (C10orf12)	^11^
PRC2.2	EZH, EED, SUZ12, RBBP	N.D.	AEBP2, JARID2	
PRC1.1	PCGF1 (RNF68, NSPC1), RING1	Noncanonical	RYBP, KDM2B (FBXL10), BCOR, SKP1	^12^
	PCGF1 (RNF68, NSPC1), RING1	Noncanonical	YAF2, KDM2B (FBXL10), BCOR, SKP1	
	PCGF1 (RNF68, NSPC1), RING1	Noncanonical	CBX8, KDM2B (FBXL10), BCOR, SKP1	
PRC1.2	PCGF2 (MEL-18), RING1	Canonical	CBX, PHC, SCMH	
PRC1.3	PCGF3 (RNF3), RING1	Noncanonical	RYBP, AUTS2, FBRS, CKII (CSNK2A1)	
	PCGF3 (RNF3), RING1	Noncanonical	YAF2, AUTS2, FBRS, CKII (CSNK2A1)	
PRC1.4	PCGF4 (BMI-1), RING1	Canonical	CBX, PHC, SCMH	
PRC1.5	PCGF5 (RNF159), RING1	Noncanonical	RYBP, AUTS2, FBRS, CKII (CSNK2A1)	
	PCGF5 (RNF159), RING1	Noncanonical	YAF2, AUTS2, FBRS, CKII (CSNK2A1)	
PRC1.6	PCGF6 (RNF134, MBLR), RING1	Noncanonical	RYBP, WDR5, L3MBTL2, ESF6-TDP1, MGA-MAX, CBX3 (HP1γ)	
	PCGF6 (RNF134, MBLR), RING1	Noncanonical	YAF2, WDR5, L3MBTL2, ESF6-TDP1, MGA-MAX, CBX3 (HP1γ)	

*N.D.* not determined.

**Table 2:** Paralogs of each subunit of PRC2 and canonical PRC1 in mammals.

Core subunit of PRC2	Homologs in fruit flies	Paralogs in mammals	Protein domain	Function	References
EED	ESC	none	WD40 repeat	Binding to H3K27me3	^9^
EZH	E(Z)	EZH1	SET domain	H3K27 methyltransferase	^17^
		EZH2			
SUZ12	SU(Z)12	none	Zinc-finger domain	DNA/RNA binding and protein–protein interaction	^17,18^
RBBP	NURF55	RBBP4	WD40 repeat	Binding to unmodified nucleosomes	^18,19^
		RBBP7			

Core subunit of canonical PRC1	Fruit fly	Mammalian	Protein domain	Function	References
RING1	RING	RING1A (RING1)	RING finger domain	Nucleosome binding and H2AK119 mono-ubiquitin ligase	^20^
		RING1B (RNF2)			
PCGF	PSC	PCGF1	RING finger domain and RAWUL domain	H2AK119 mono-ubiquitin ligase and protein–protein interaction	^21^
		MEL18 (PCGF2)			
		PCGF3			
		BMI1 (PCGF4)			
		PCGF5			
		PCGF6			

In addition to the core subunits of PRC2, several other proteins can bind to these subunits and modulate the enzyme activity of PRC2. Two different types of PRC2 complexes (PRC 2.1 and PRC 2.2) have been identified based on their noncore subunit proteins in humans (Table 1)^11,12,27^. PRC2.1 contains three other subunits, including polycomb-like protein (PCL), PRC2-associated LCOR isoform (PALI), elongin B/C and PRC2-associated protein (EPOP) (Table 1)^11,12,28–30^. PCL has three paralogs: PCL1, PCL2, and PCL3. They are also known as PHF1 (PCL1), MTF2 (PCL2), and PHF19 (PCL3), respectively. PALI, also known as C10ORF12, has two paralogs: PALI1 and PALI2^28–30^. Three noncore subunit proteins (PCL, PALI and EPOP) can act as enhancers to facilitate the catalytic activity of PRC 2.1. The function of PCL is essential for H3K27me3 by PRC 2.1 because the recognition of H3K36me2/3 by the TUDOR domain of PCL is a prerequisite for PRC 2.1 to introduce H3K27me3 marks^31^. PCL is also required for the recognition of unmethylated CpG islands of DNA by PRC 2.1^32,33^. PALI1 can facilitate the catalytic activity of PRC2 both in vitro and in vivo^34^. Similar to the phenotype of EZH2-deficient mice, PALI1-deficient mice exhibit embryonic lethality^34^. EPOP can mediate the interaction between PRC2.1 and elongin B/C, which is important for maintaining the transcriptional repression of PRC2’s target locus^35^.

Adipocyte enhancer-binding protein 2 (AEBP2) and Jumonji AT-rich interactive domain 2 (JARID2) are additional subunits that for PRC 2.2 along with the PRC2 core subunits (Fig. 1 and Table 1)^11,12,27,36^. Both AEBP2 and JARID2 are required to recruit PRC 2.2 to chromatin by specifically binding to the CpG-rich region of DNA^36^. Recent studies have indicated that Jarid2-containing PRC 2.2 can specifically recognize and bind to the mono-ubiquitinated lysine 119 residue at histone H2A (H2AK119Ub) tagged by the PRC1.1 (noncanonical PRC 1) complex (Fig. 1b)^37^. The binding of H2AK119Ub by Jarid2 can further facilitate the methyltransferase activity of PRC 2.2 (Fig. 1b)^37^.

The subunits of PRC1 complexes are much more diverse than those of PRC2 (Fig. 1b, Table 1)^11,12^. There are two groups of PRC1 complexes categorized based on the original findings in fruit flies. Canonical PRC1 complexes are composed of subunit proteins conserved from flies to mammals, whereas the subunit proteins of noncanonical PRC1 complexes are less conserved in flies^38^. Really interesting new gene 1 (RING) and polycomb group ring finger (PCGF) have been found in both canonical and noncanonical PRC1 complexes, suggesting that these proteins are structurally and functionally essential components^38^. RING proteins exhibit two paralogs (RING1A and RING1B) that possess E3 ubiquitin ligase activity when they are combined with PCGF proteins (H2AK119Ub activity) (Fig. 1, Tables 1 and 2)^9,11,12,17–21,38,39^. PCGF proteins exhibit six paralogs (PCGF1–PCGF6)^20^. Upon interaction with RING proteins, PCGF proteins can increase ubiquitin ligase activity by acting as cofactors^39,40^. Each PCGF paralog (PCGF1 through PCGF6) can be a subunit of different types of PRC1 complexes (PRC1.1 through PRC 1.6) (Fig. 1a, Tables 1 and 2)^9,11,12^.

Among the six different types of PRC1 complexes (PRC1.1–PRC 1.6), the PCGF2 (MEL-18)-containing PRC1.2 and PCGF4 (BMI-1)-containing PRC1.4 complexes are classified as canonical PRC1 complexes^41^. Chromobox homologs (CBX) can form canonical PRC1 complexes with RING proteins and PCGF2 (MEL-18) or PCGF4 (BMI-1) (Fig. 1a)^41^. Five CBX paralogs (CBX2, CBX4, CBX6, CBX7, CBX8) have been found to act as subunits of the canonical PRC1 complex in mammals (Fig. 1a)^41^. The proposed role of CBX in the canonical PRC1 complex is to recruit PRC1 to H3K27me3 tags because CBX proteins contain chromodomains that recognize the H3K27me3 tag introduced by PRC2 (Fig. 1a)^42,43^. Additionally, polyhomeotic homolog (PHC) and sex comb on midleg homolog (SCMH) can interact with core proteins (RING and PCGF) to form canonical PRC1 complexes (Fig. 1a, Table 1)^44,45^. PHC proteins exhibit three paralogs (PHC1-PHC3)^3,7^. SCMH proteins exhibit two paralogs (SCMH1 and SCMH2)^46^. Both types of proteins contain a sterile α motif domain that allows them to bind to other canonical PRC1 complex proteins and participate in the recruitment of PRC1 to chromatin (Table 1)^44,45^. PHC proteins also have zinc-finger domains that facilitate nucleic acid binding and chromatin compaction^46^.

The noncanonical PRC1 complex is composed of more protein subunits (Table 1)^11,12^. In the noncanonical PRC1 complex, the core subunits (RING1 and PCGF) can interact with ring and YY1 binding protein (RYBP) or YY1-associated factor 2 (YAF2) or CBX8 (Table 1) via *C*-terminal ring finger and WD40 ubiquitin-like (RAWUL) domains^12,47^. Previous observations have indicated that RYBP can compete with CBX for the binding site of RING1B^48^. YAF2 and RYBP occur in the noncanonical PRC1 complex in a mutually exclusive manner, since YAF2 is a homolog of RYBP (Table 1).

The function of the noncanonical PRC1 complex is clearly different from that of the canonical PRC1 complex (Fig. 1). According to the ‘hierarchical repressive model’, PRC2 can repress a target locus via an H3K27me3 tag. The canonical PRC1 complex can recognize this methylation tag through CBX and further repress a target locus by introducing a H2AK119 mark (Fig. 1a)^43^. Recently, the RYBP-containing noncanonical PRC1 complex has been found to show higher E3 ligase activity than PCGF4-RING1B containing canonical PRC1 complex^49^. This finding suggests that another pathway for transcriptional repression exists in addition to the ‘hierarchical repressive model’. Indeed, the CxxC DNA-binding domain of KDM2B in the PRC1.1 complex can specifically recognize CpG DNA sequences and recruit PRC1.1 to a target locus^50,51^. PRC1.1 then suppresses specific mRNA transcription via an H2AK119ub tag (Fig. 1b)^50,51^. Thereafter, PRC 2.2-containing Jarid2 can specifically recognize and bind the H2AK119Ub tag and further modify the structure of chromatin by introducing an H3K27me3 tag (Fig. 1b)^37,50,51^. This model is known as the ‘reverse hierarchical repressive model’ because PRC1.1 first represses the specific transcription of mRNA instead of PRC2.2.

In fruit flies, putative DNA regions recognized by PRCs have been identified, validated, and designated as PcG/trithorax-group response elements (PREs)^2–7^. The existence of vertebrate PRE sites around CpG-rich sequences has also been suggested^36,52^. However, the conserved DNA-binding motif of mammalian PRCs and the detailed mechanism by which mammalian PRCs recognize specific DNA regions remain elusive. In fruit flies, it has been suggested that the pleiohomeotic (Pho) protein can recognize PREs and guide the core subunits of PRC1 and PRC2 to PREs since the core subunits of PRC2 or PRC1 do not directly bind to DNA^53^. In vertebrates, YinYang1 (YY1), a Pho homolog, can bind to a conserved DNA region and interact with PRC1 subunits^54^. Therefore, YY1 may recognize PRE sites and guide noncanonical PRC1 by interacting with RYBP or YAF2^55^.

## The role of PcG proteins in immune regulation

**Fig. 2:**
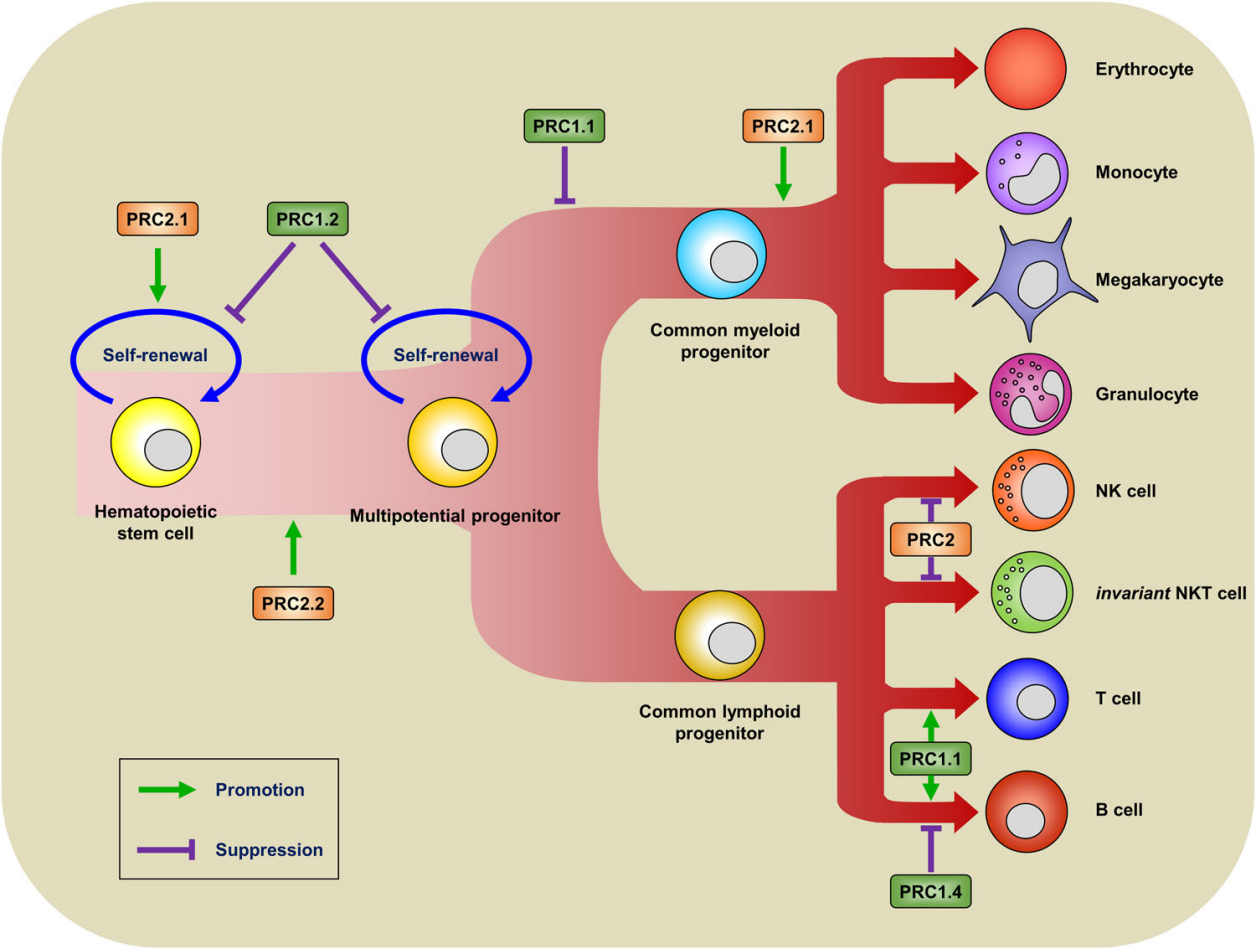
Functional contribution of PcG proteins during immune cell differentiation. Schematic representation of particular PRC2 or PRC1 complexes involved in hematopoiesis according to the ‘hierarchical repressive model’.

A knockout (KO) mouse model and the cell type-specific deletion of PcG genes generated in a conditional knockout (cKO) mouse model using the cre-lox system have been used in most studies to study the function of PcG proteins in immune regulation (Table 3)^56–82^. Except gene encoding RBBP, mice deficient in the genes encoding each core subunit of PRC2 have been generated and characterized (Table 3)^56–82^. Based on animal studies, Ezh1 can partially replace the function of EZh2 in specific cell types^83,84^. For example, Ezh2 is not required for the self-renewal activity of long-term hematopoietic stem cells (LT-HSCs) in adult bone marrow^64^. However, Ezh1-deficient mice exhibit immunodeficiency due to a significant loss of the self-renewal activity of HSCs^56^. Because the *INK4a*/*Arf* locus, encoding *p16INK4a* and *p19Arf*, which can suppress cell cycle progression, is a target of PcG-mediated repression, the deficiency of certain core subunits of PRC2 and canonical PRC1 can cause the loss of self-renewal activity of HSCs^85^. In addition to EZH1 deficiency, insufficiency of other subunits of PRC2, including EED or SUZ12, can lead to the loss of the self-renewal activity of HSCs^64,66^. The deficiency of some canonical subunits of PRC1, including BMI-1 and PHC1, can also cause the loss of self-renewal activity of HSCs^67,68,75^. However, other canonical subunits of PRC1, including MEL18, CBX2, CBX8, and PHC2, do not influence the self-renewal activity of HSCs^72,76,77,79^. These phenotypic variations observed in each of the mice deficient in different PcG subunits reflect structural heterogeneity depending on the specific stage of cells or tissues due to the redundancy or paralogs of each PcG subunit (Table 3). Cell type-specific roles of various PRC1 and PRC2 complexes have already suggested (Fig. 2)^12^. In support of these ideas, EZH2 expression in LT-HSCs peaks on embryonic day 14.5 and gradually decreases thereafter until 10 months postnatal^64^. However, EZH1 expression in LT-HSCs gradually increases from embryonic day 14.5 to 10 months after birth^64^. BMI-1 and MEL18 expression patterns also follow the paradigm of EZH1/2 expression. BMI-1 is mainly expressed in specific lineage precursors of immune cells, whereas the expression of MEL-18 is correlated with mature immune cell populations^86^. In addition to contributing to the self-renewal activity of HSCs, PcG proteins participate in the differentiation of hematopoietic progenitor cells (HPCs) into specific lineages of immune cells. The contributions of PRC2 and canonical PRC1 to immune cell differentiation according to the ‘hierarchical repressive model’ are summarized in Table 3 and Fig. 2.

**Table 3:** Hematopoietic cell fate decision by PRC2 and canonical PRC1 according to the ‘hierarchical repressive model’ in mice.

Complex	Gene	mouse type	Abnormality of specific gene expression	Phenotype in mice	Reference
PRC2	Ezh1	KO	*Gata3, Runx1, Meis1, Myb Dntt, Flk2, Igh6*, and *Ikaros*	Loss of self-renewal activity of HSC and defective in B cell development	^56^
	Ezh2	KO	None	Embryonic lethality at embryonic day 7.5	^57^
		cKO (Tie2-Cre)	*Gata2*	Embryonic lethality due to fatal anemia	^58^
		cKO (ERT-Cre)	*Gata2*	Defects in B cell development	^58^
		cKO (CD4-Cre)	*T-bet, Eomes*, and *Gata3*	Enhanced Th cell plasticity (spontaneous Th_1_ or Th_2_ polarization without stimulation)	^59^
		cKO (CD4-Cre)	*Ifng*, *Gata3*, and *Il10*	Increased apoptosis of effector Th_0_ cells	^60^
		cKO (GzmB-Cre)	*Foxo1*	Fewer antigen-specific CD8^+^ T cells	^61^
		cKO (Vav1-Cre)	*Klrk1, Il2ra, Il7r, Cxcr3, Ccr7*, and *Xcr1*	Increased number of NK cells	^62^
		cKO (CD4-Cre)	*Cd160, Zbtb16, Ccr4, Il4, Ifng*, and *Il12*	Increased number of *invariant* NKT cells	^63^
	Eed	KO	*Ckdn2a, Cdkn1a, Cdkn2b, HoxC4, p21*, and *Wig1*	Severe anemia and leukopenia	^64^
		cKO (Vav1-Cre)	*Ckdn2a, Cdkn1a, Cdkn2b, HoxC4, p21*, and *Wig1*	Pancytopenia without defect of hematopoietic stem cell (HSC)	^64^
		cKO (Mx1-Cre)	None	Higher frequency of spontaneous T cell leukemia	^65^
		cKO (CD4-Cre)	*Foxo1*	Fewer antigen-specific CD8^+^ T cells	^61^
	Suz12	cKO (Vav1-Cre)	None	Loss of HSC function and defects in lymphocyte development	^66^
Canonical PRC1	Bmi1	KO	None	Less proliferative activity of leukemic stem and progenitor cells	^67^
		KO	*p16, p19, Wig1, Tjp1*, and *Hoxa9*	Loss of self-renewal activity of HSC	^68^
		KO x OTII	*Ink4a/Arf, Bax, Puma, Noxa, Bad*, and *Fas*	Increased apoptosis of memory Th_2_ cells	^69^
		KO	*Cdkn2a*	Impaired cell expansion of double negative (DN) thymocytes	^70^
		KO	*Cdkn2a*	Decreased Th_2_ cell polarization	^71^
	Mel18	KO	None	No significant defect of HSC function	^72^
		KO	*Gata3*	Impaired Th_2_ cell polarization	^73^
	Phc1	KO	None	Perinatal lethality	^74^
		Reconstitution of E14.5 HSC with KO	None	Reduced number of lymphocytes	^75^
	Phc2	KO	*Vcam1*	Defective mobilization of hematopoietic stem and progenitor cell (HSPC)	^76^
	Cbx2	KO	None	Reduced number of thymocytes and splenocytes without defect of HSC and defective in T cell development	^77^
	Cbx4	KO	None	Thymic hypoplasia	^78^
		cKO (Foxn1-Cre)	*K14, Cd80*, and *Cd86*	Thymic hypoplasia	
		cKO (Lck-Cre)	None	No apparent defects	
	Cbx8	cKO (ERT-Cre)	*Hoxa9*	No apparent defects	^79^
		cKO (Cγ1-Cre)	*Cdkn1a, Prdm1*, and *Irf4*	Defects in germinal center formation	^80^
	Ring1B	cKO (Mx1-Cre)	*Cdkn2a* and *Ccnd2*	Hypocellular bone marrow	^81^
		cKO (ER^T2^-Cre)	*Cdkn2a* and *Ccnd2*	Hypocellular bone marrow	
	Rng1A and Ring1B	Double cKO (Lck-Cre)	*Pax5, Ebf1, Irf4, and Irf8*	Defects in B cell development	^82^

*KO* knockout mice, *cKO* conditional knockout mice.

Studies on the importance of PcG proteins in immune cell function are much less common than studies on the influence of PcG proteins during the differentiation of immune cells (Table 3). Most studies on the functional contribution of PcG proteins to immune cell function have focused on T cell function (Table 3). The CD8^+^ T cell-specific deletion of *Ezh2* or *Eed* using the CD4-Cre or granzyme B-Cre system revealed that the antigen-specific activation of CD8^+^ T cells requires the function of the PRC2 complex (Table 3)^61^. Interestingly, the contribution of PcG proteins to CD4^+^ T cell function is controversial because the phenotypes of each of the PcG protein-deficient mice are quite different from each other. For example, CD4^+^ T cell-specific *Ezh2* deletion has led to type 2 helper T cell (Th_2_)-prone immunity via the accumulation of memory Th_2_ cells, which exacerbates allergic diseases (Table 3)^59^. However, *Bmi1* and *Mel18* knockout mice are defective in Th_2_ cell differentiation^71,73^. Furthermore, *Bmi1* knockout mice exhibit the enhanced apoptosis of memory Th_2_ cells^69^.

## Current RNA-seq and chromatin immunoprecipitation (ChIP)-seq data for identifying the target loci of PcG proteins

The phenotypic analysis of transgenic mice in combination with RNA-seq and chromatin immunoprecipitation (ChIP)-seq analyses might be a good approach for identifying additional target loci of PcG proteins or the unique functions of each PcG protein paralog in specific immune cell types. Table 4 summarizes current gene chip and RNA-seq databases generated from specific cell types of transgenic mice or specific cell lines subjected to the inhibition of PcG function (http://www.ebi.ac.uk/arrayexpress/). Most of the RNA-seq data were acquired from embryonic stem cells (ES cells), HSCs (LSK cells, LSK, Lin^−^Sca-1^+^c-kit^+^ cells), hematopoietic stem and progenitor cells (HSPC), and cancer cell lines including leukemia, multiple myeloma, sarcoma, ovarian tumor, and gastric cancer cell lines because the initial identification of PcG function emphasized the maintenance of self-renewal activity (Table 4). To expand the collection of differentially expressed gene (DEG) data, RNA-seq analyses need to be performed using a broad range of immune cells, including B cells, monocytes, dendritic cells, mast cells, and polymorphonuclear cells. All DEGs identified in PcG-defective cells might not be direct targets of PcG proteins. Chip-seq data might be needed to verify whether these DEGs are direct targets of PcG proteins. Table 5 summarizes the current ChIP-seq databases for specific cell types (http://www.ebi.ac.uk/arrayexpress/). The DNA-binding sites of most core subunits of PRC2, except for RBBP, and the core subunits of PRC1.2 (RING1B and MEL18) have been analyzed by ChIP-seq (Table 5). The DNA-binding sites of some paralogs of CBX and Jarid2, a subunit of PRC2.2, have also been analyzed (Table 5). However, most of the ChIP-seq data were acquired from stem cell lineages with few exceptions (Table 5). Therefore, a broad range of cells need to be analyzed by Chip-seq using antibodies against the remainder of the PcG proteins, including RBBP, BMI-1, and PHC, to identify novel target genes repressed by PcG proteins.

**Table 4:** RNA-seq or gene-chip data from loss or gain of function of each PcG subunit protein^a, b^.

Unit	Data ID	Target cell (cell line)	Functional alteration (method)
EZH1	E-GEOD-36288	LSK (Lin^−^Sca-1^+^c-kit^+^) cells	Loss of function (KO)
	E-GEOD-59090	CD34^+^ hematopoietic stem and progenitor cell (HSPC)-derived proerythroblasts	Loss of function (shRNA)
EZH1/ EZH2	E-GEOD-62198	MLL-AF9 induced leukemia progenitor	Loss of function (inhibitor, UNC1999)
EZH2	E-GEOD-59090	CD34^+^ HSPC-derived proerythroblasts	Loss of function (shRNA)
	E-GEOD-71870	Ovarian tumor (OC8 cell line)	Loss of function (shRNA)
	E-GEOD-71671	Monocyte (THP-1 cell line)	Loss of function (inhibitor, GSK126)
	E-MTAB-3227	Gastric cancer (MKN45 cell line)	Loss of function (siRNA)
	E-GEOD-82072	Megakaryocyte-erythrocyte precursor	Loss of function (cKO; scl-cre)
	E-GEOD-82073	Long-term-hematopoietic stem cell (LT-HSC)	Loss of function (cKO; scl-cre)
	E-MTAB-2893	Chronic myeloid leukemia CD34^+^ cells	Loss of function (inhibitor, GSK343)
	E-MTAB-3552	Nonchronic myeloid leukemia CD34^+^ cells	Loss of function (inhibitor, GSK343)
	E-MTAB-5766	Acute myeloid leukemia cell (F-36P, MOLM-13, and OCI-M2 cell line)	Loss of function (shRNA)
	E-MTAB-7739	Macroglobulinemia cell (RPCI-WM1 cell line)	Loss of function (inhibitor, Tazemetostat)
	GSE101316	Mouse bone marrow-derived macrophage	Loss of function (KO)
EED	E-GEOD-12982	Embryonic stem cell (ES cell)	Loss of function (KO)
	E-GEOD-49305	ES cell	Loss of function (KO)
	E-GEOD-62198	MLL-AF9 induced leukemia progenitor	Loss of function (shRNA)
	E-GEOD-53508	ES cell	Loss of function (KO)
	E-GEOD-53506	ES cell	Loss of function (KO)
	E-GEOD-59090	CD34^+^ HSPC-derived proerythroblasts	Loss of function (shRNA)
SUZ12	E-GEOD-31354	ES cell	Loss of function (KO)
	E-GEOD-53508	ES cell	Loss of function (Genetrap)
	E-GEOD-59090	CD34^+^ HSPC-derived proerythroblasts	Loss of function (shRNA)
	E-GEOD-60808	HSPC	Loss of function (shRNA)
Jarid2	E-GEOD-60808	HSPC	Loss of function (shRNA)
RING1A	E-MTAB-5661	ES cell	Loss of function (KO)
RING1A/B	E-MTAB-5661	ES cell	Loss of function (KO, cKO)
RING1B	E-GEOD-67868	ES cell	Loss of function (KO)
	E-GEOD-69824	ES cell	Loss of function (KO)
	E-GEOD-71007	Ewing’s sarcoma (A4573, A673, ES1, and TC71 cell line)	Loss of function (shRNA)
Mel18	E-GEOD-67868	Embryonic stem cell	Loss of function (shRNA)
Bmi1	E-GEOD-21912	Multiple myeloma(RPMI-8226 cell line)	Loss of function (shRNA)
	E-GEOD-19796	HSPC	Loss of function (KO)
	E-GEOD-20958	ES cell and HSC	Gain of function (overexpression)
	E-GEOD-31086	Common myeloid progenitor	Loss of function (KO)
	E-GEOD-54262	Erythroleukemia (K562 cell line) and Chronic myeloid leukemia	Loss of function (shRNA)
	E-GEOD-71007	Ewing’s sarcoma (A673 and TC71 cell line)	Loss of function (shRNA)
Cbx7	E-GEOD-34191	ES cell	Loss of function (shRNA)
PCGF1	E-GEOD-33280	HSPC	Loss of function (shRNA)
PCGF3/5	E-MTAB-5642	ES cell	Loss of function (KO)

*KO* knockout mice, *cKO* conditional knockout mice.

^a^Information in Table 4 was acquired from https://www.ebi.ac.uk/arrayexpress/.

^b^Each reference for Table 4 is contained within contents of each Data ID.

**Table 5:** ChIP-seq data of each PcG subunit protein^a, b^.

Unit	Data ID	Target cell (cell line)
EZH1	E-GEOD-59090	CD34^+^ hematopoietic stem and progenitor (HSPC)-derived proerythroblasts
EZH2	E-GEOD-18776	Mouse embryonic stem cell (ES cell)
	E-MTAB-1305	Human ES cell
	E-GEOD-51079	In vitro cultured Th_1_ and Th_2_ cell
	E-GEOD-42706	mouse resting B cell (CD43^-^ B cell)
	E-GEOD-49178	ES cell
	E-GEOD-52300	Human liver cancer (HepG2 cell line)
	E-GEOD-48518	induced pluripotent stem cell
	E-GEOD-46536	ES cell
	E-GEOD-53495	Human embryonic kidney cell (293T Rex cell line)
	E-GEOD-57632	Multiple myeloma
	E-GEOD-61586	Neural stem cell and glioma (SF7761 cell line)
	E-MTAB-2002	Mouse ES cell
	E-GEOD-47082	Mouse ES cell
	E-GEOD-59090	CD34^+^ HSPC-derived proerythroblasts
	E-GEOD-70440	Mammary gland
	E-GEOD-60160	Mouse ES cell
	E-MTAB-6410	Chronic lymphocytic leukemia
	GSE101320	Mouse bone marrow-derived macrophages
EED	E-GEOD-61902	Spermatocyte
	E-GEOD-59090	CD34^+^ HSPC-derived proerythroblasts
	E-MTAB-6165	Mouse ES cell
SUZ12	E-GEOD-34483	Mouse ES cell
	E-GEOD-42616	Mouse ES cell
	E-GEOD-44286	Mouse ES cell
	E-GEOD-52300	Human liver cancer (HepG2 cell line)
	E-MTAB-2481	Mouse ES cell
	E-GEOD-55698	ES cell
	E-GEOD-52619	Mouse ES cell
	E-GEOD-47528	Primary CD4^+^ helper T cell
	E-GEOD-58023	ES cell
	E-GEOD-62437	MLL-AF9 transformed leukemia
	E-GEOD-43915	Mouse E13.5 brain
	E-GEOD-59090	CD34^+^ HSPC-derived proerythroblasts
	E-GEOD-57926	Mouse heart and embryonic fibroblast cell
	E-GEOD-61148	Mouse thymocyte and thymic T cell tumor (ILC87 cell line)
	E-GEOD-74330	Mouse ES cell
	E-GEOD-83082	Mouse ES cell
Jarid2	E-GEOD-19708	Mouse ES cell
RING1B	E-GEOD-23716	Mouse ES cell
	E-GEOD-55698	ES cell
	E-GEOD-43915	Mouse E13.5 brain
	E-GEOD-67868	Mouse ES cell
	E-GEOD-72164	Mouse ES cell
	E-GEOD-74330	Mouse ES cell
MEL18	E-GEOD-67868	Mouse ES cell
	E-GEOD-74330	Mouse ES cell
CBX2	E-GEOD-29611	Erythroleukemia (K562 cell line)
CBX3	E-GEOD-29611	Erythroleukemia (K562 cell line)
	E-GEOD-32465	Human colon cancer (HCT116 cell line) and erythroleukemia (K562 cell line)
	E-GEOD-28115	Human colon cancer (HCT116 cell line)
	E-GEOD-44242	Mouse ES cell and induced pluripotent stem cell
CBX7	E-GEOD-23716	Mouse ES cell
	E-GEOD-42466	Mouse ES cell
	E-GEOD-42706	mouse resting B cell (CD43^-^ B cell)
CBX8	E-GEOD-29611	Erythroleukemia (K562 cell line)
	E-GEOD-54052	Mouse ES cell

^a^Information in Table 5 was acquired from https://www.ebi.ac.uk/arrayexpress/.

^b^Each reference for Table 5 is contained within contents of each Data ID.

## Therapeutic agents for treating hematopoietic malignancies by inhibiting the activity of PcG proteins

Since the function of PcG proteins is important to maintain the self-renewal activity of stem cells, PcG proteins might act as oncogenes to facilitate tumorigenesis. In support of this idea, high expression of EZH2 has been observed in several hematopoietic malignancies, including myelodysplastic syndromes, acute myeloid leukemia, and various types of lymphomas^87–89^. In particular, EZH2 deficiency in mice can inhibit leukemogenesis by decreasing the proliferation rate of leukemia^90^. Consistent with these observations, the expression levels of canonical subunits of PRC1, including BMI1, CBX7, CBX8, and RING1A, are elevated in many hematopoietic-originating tumors^88,91,92^. A mouse model involving *Bmi1*-deficient mice with transformed cells also supports the notion that BMI1 can act as an oncogene in some hematopoietic malignant cells^93^. However, the loss of function of PcG proteins by mutation or deletion might also cause hematopoietic malignancies^91^. In particular, defects in core subunits of PRC2, including EZH2, EED, and SUZ12, have been found in various acute lymphoblastic leukemia and myelodysplastic syndromes^94–97^. Therefore, at least PRC2 can act as an oncogene or a tumor suppressor depending on the type of hematopoietic malignant cells involved^91^. Further study is needed to define the mechanisms underlying the dual functions of these proteins in tumorigenesis.

Table 6 summarizes the inhibitors of PcG proteins applied to clinical trials in hematopoietic malignancies and other types of tumors. Major groups of inhibitors target EZH enzyme activity (Table 6). Most EZH2 inhibitors undergoing clinical trials compete with SAM for binding to the SET domain^98^. Among the competitive inhibitors of EZH, tazemetostat (EPZ-6438), an orally administered small chemical, has been applied to a broad range of malignant cell types, including lymphoma, sarcoma, mesothelioma, ovarian cancers and advanced solid tumors (Table 6)^98^. Other inhibitors of PcG proteins that are currently undergoing clinical trials target EED and BMI-1 activity (Table 6). MAK683 is an allosteric EED inhibitor that drives conformational changes in the H3K27me3-binding pocket of EED upon binding^99^. These conformational changes in EED further prevent the interaction between EED and EZH2, thus blocking H3K27me3^99^. PTC596 is a BMI-1 inhibitor that can facilitate the degradation of BMI-1 by inducing the cyclin-dependent kinase 1-mediated biphosphorylation of the *N*-terminus of BMI-1^100^.

**Table 6:** Inhibitors of PcG proteins undergoing current clinical trials in malignant cells^a^.

Target polycomb subunit	Agent	Mode of action	NCT ID	Phase	Target tumor types	Status
EZH2	Tazemetostat (EPZ-6438)	S-adenosyl- _L_-methionine (SAM) competitive inhibitor	NCT02875548	II	Diffuse large B cell lymphoma	Recruiting
			NCT02601950	II	INI (hSNF5; SMARCB1)-negative tumor and relapsed/refractory synovial sarcoma	Recruiting
			NCT01897571	I/II	Advanced solid tumor and B cell lymphoma	Active, not recruiting
			NCT02601937	I	INI (hSNF5; SMARCB1)-negative tumor and synovial sarcoma	Recruiting
			NCT02860286	II	Malignant mesothelioma	Completed
			NCT03213665	II	Relapsed/refractory advanced solid tumor and non-Hodgkin’s lymphoma	Recruiting
			NCT02889523	Ib/II	Diffuse large B cell lymphoma	Suspended
			NCT03217253	I	Metastatic or unresectable solid tumor	Active,not recruiting
			NCT03348631	II	Recurrent ovarian cancer	Suspended
	GSK2816126	SAM competitive inhibitor	NCT02082977	I	Relapsed/refractory diffuse large B cell lymphoma and transformed follicular lymphoma	Terminated
	CPI-1205	SAM competitive inhibitor	NCT02395601	I	B cell lymphoma	Completed
	CPI-0209	Second generation inhibitor of EZH2	NCT04104776	I/II	Advanced solid tumor	Recruiting
	PF-06821497	SAM competitive inhibitor	NCT03460977	I	Relapsed/refractory small cell lung cancer,castration-resistant prostate cancer, and follicular lymphoma	Recruiting
	SHR2554	SAM competitive inhibitor	NCT03603951	I	Relapsed/refractory mature lymphoid neoplasm	Recruiting
EZH1 and EZH2	DS-3201b (Valemetostat tosylate)	SAM competitive inhibitor	NCT04102150	II	Relapsed/refractory adult T cell leukemia/lymphoma	Recruiting
EED	MAK683	Binds to EED and change overall shape of PRC2	NCT02900651	I/II	Diffuse large B cell lymphoma	Recruiting
BMI1	PTC-596	Phosphorylation of BMI-1 at two *N*-terminal sites which leads to the degradation of BMI-1	NCT03605550	Ib	High grade glioma and diffuse intrinsic pontine glioma	Recruiting
			NCT03206645	I	Ovarian cancer	Recruiting
			NCT02404480	I	Advanced solid cancer	Completed
			NCT03761095	I	Leiomyosarcoma	Recruiting

^a^Information in Table 6 was acquired from https://clinicaltrials.gov/.

## Conclusion and future prospects

In this review, we highlighted the structural diversity of mammalian PRC2 and PRC1 complexes related to their functional contribution to immune regulation. We also described currently available RNA-seq and ChIP-seq data that could be used to mine new target loci of PcG proteins. Finally, we listed the PcG inhibitors currently undergoing clinical trials. Many previous reports have demonstrated that PcG proteins are major chromatin modifiers that can modulate many biological processes by influencing specific gene repression, mainly using loss-of-function models.

Unfortunately, we still do not know how many different types of PRCs exist in nature due to structural heterogeneity caused by many paralogs and accessory proteins recruited by PRC complexes. We also do not know how each different PRC containing a particular paralog as a subunit contributes to the phenotype of a specific cell type. Solving these unknown issues might provide novel targets for PcG-mediated gene regulation and expand the range of PcG proteins considered as therapeutic targets to treat other human diseases in addition to cancer.
